# Features of the Metabolisms of Cardiac Troponin Molecules—Part 1: The Main Stages of Metabolism, Release Stage

**DOI:** 10.3390/cimb44030092

**Published:** 2022-03-20

**Authors:** Aleksey Michailovich Chaulin

**Affiliations:** 1 Department of Cardiology and Cardiovascular Surgery, Medical Faculty, Samara State Medical University, 443099 Samara, Russia; a.m.chaulin@samsmu.ru; 2 Department of Clinical Chemistry, Samara Regional Clinical Cardiological Dispensary, 443070 Samara, Russia

**Keywords:** molecules, troponin T, troponin I, metabolism, stages of metabolism, release stage, diurnal rhythm, diagnosis

## Abstract

Cardiac troponins (cTns) have long been the most valuable and specific biomarkers for detecting ischemic myocardial cells (MCs) injury, which is one of the key signs of myocardial infarction (MI). Modern methods (highly sensitive and ultra-sensitive immunoassays (hs-cTns)) of detection are an important and indispensable tool for the early diagnosis of MI and the choice of patient management protocols. Timely diagnosis of MI can significantly improve the prognosis of patients. However, in real clinical practice, doctors often face a significant problem when using cTns—the difficulty of differential diagnosis due to frequent and unexplained increases in the concentration of cTns in blood serum. In addition, there is conflicting information that may potentially affect the diagnostic capabilities and value of cTns: the influence of certain biological factors (diurnal rhythm, gender and age) on serum cTns levels; extra-cardiac expression of cTns; the possibilities of non-invasive diagnosis of MI; and other pathological conditions that cause non-ischemic injury to MCs. To solve these problems, it is necessary to concentrate on studying the metabolism of cTns. The review of our current knowledge about cTns metabolism consists of two parts. In this (first) part of the manuscript, the main stages of cTns metabolism are briefly described and the mechanisms of cTns release from MCs are considered in detail.

## 1. Introduction

Cardiac troponins (cTns) (cTnI and cTnT) have long been the most valuable and specific biomarkers for detecting ischemic myocardial cells (MCs) injury, which is one of the key signs of myocardial infarction (MI) [1,2,3].

According to current studies, cTn molecules are detectable in blood serum [1,2,3,4,5] and a number of other body fluids such as urine [6,7] and oral fluid [8,9,10] of almost all healthy people. This has been made possible by the development and use of high sensitivity and ultra-sensitivity assay methods (hs-cTns) that can detect very low concentrations of cTns (at a few ng/L or less) in human body fluids [10,11]. This ability to detect low levels of cTns has improved the early diagnosis of MI by detecting these cardiac markers in the first hours from the onset of the clinical picture of MI. Thus, due to a number of large and multicenter studies, it was possible to validate the early diagnostic algorithms of exclusion/confirmation of MI without ST-segment elevation (NSTEMI) (0–1 h and 0–2 h algorithm), which are reflected in the current recommendations of the European Society of Cardiology. The basic principle of these algorithms is based on the assessment of the kinetics of hs-cTns levels during the first hour (0–1 h algorithm) and during two hours (0–2 h algorithm) [12]. However, there are different optimal and threshold concentrations for each high-sensitivity immunoassay (developed by different manufacturers).

In accordance with current guidelines for the management of patients with NSTEMI, MI is suspected based on the patient’s history, symptoms of myocardial ischemia, and electrocardiographic (ECG) signs of ischemia. cTns value and dynamics lead to the triage decision. Cardiac ultrasound and coronary angiography (CAG) are further explorations for the final verification of the diagnosis and the choice of treatment protocol [13].

As a rule, the diagnosis of NSTEMI is excluded when cTns levels are low or very low on admission to the emergency department and when cTn levels do not increase on repeat collection and blood testing after 1 or 2 h. The management protocol for these patients is early discharge and subsequent outpatient or elective treatment if necessary. In cases where cTn levels are above the accepted limit (for the appropriate high-sensitivity immunoassay and the company) and a repeat blood draw and test shows a significant increase in cTn levels, the likelihood of a diagnosis of NSTEMI is high. The need to use other diagnostic methods is determined by the fact that cTn levels may be elevated not only in MI but also in quite a number of pathologies (e.g., sepsis, acute myocarditis, hypertensive crisis, pulmonary embolism (PE), heart arrhythmias, cardiomyopathies (CMPs), chronic kidney disease (CKD), heart failure (HF), cardiotoxic effects of some chemotherapeutic agents etc.) [13,14,15,16,17,18,19,20,21,22,23,24,25,26] and physiological conditions (for example, long, heavy, and strenuous exercise and/or psycho-emotional stress) [27,28,29,30] which affect MCs and contribute to cTns release into blood serum by some mechanisms. The mechanisms of MCs injury in these conditions differ from the main mechanism which is characteristic of MI—ischemic necrosis of MCs. Considering this circumstance, cTns cannot be considered as absolute specific biomarkers of MI but can be considered as absolute specific biomarkers of “myocardial injury”. A number of clinical studies also indicate that, in most cases, serum levels of cTns are elevated in pathologies which are not associated with MI [31,32,33,34,35]. For example, according to a large study that included 1573 patients with elevated levels of cardiac troponin T (cTnT), MI was confirmed in only 10% of them. The remaining patients (about 90%) with elevated levels of cTnT were diagnosed with other pathologies that caused MCs injury and increased cTn levels by non-ischemic mechanisms. In addition, a very interesting finding in this study is that in approximately 30% of patients with elevated serum cTnT levels, the exact cause and mechanism of the elevation could not be determined [35]. Thus, elevated cTns levels may indicate the diagnosis of MI only if there are any clinical signs of myocardial ischemia and typical myocardial ischemic changes of ECG, whereas without signs of ischemia cTns merely indicate the presence of MCs injury.

In accordance with the main guidance document (Fourth Universal Definition of MI), the main criteria for MI are the following: (1) myocardial injury detected using cTns; (2) symptoms of myocardial ischemia; (3) ischemic changes on ECG and, in particular, the appearance of a pathological Q wave; (4) the identification of areas of non-viable myocardium using imaging methods; and (5) the detection of a blood clot in the coronary arteries using CAG or autopsy. The term “myocardial injury” should be used when there is evidence of elevated cTn values (>99th percentile). The myocardial injury is considered acute if there is a rise and/or fall in cTn values [36]. If the levels of cardiac troponins are constantly elevated, then this is a chronic myocardial injury. This is most often associated with structural heart diseases (CMPs, HF) or CKD.

Myocardial injury and myocardial infarction are different forms of pathology. So, if serum levels of cTns are elevated, but there are no signs of myocardial ischemia, then this is myocardial injury. If elevated serum levels of cTns are combined with signs of myocardial ischemia, then this is a MI [36].

In addition, a number of researchers are actively considering the possibility of using other body fluids (e.g., urine and oral fluid) for diagnosis and further prognosis assessment for patients suffering from cardiovascular diseases (CVD) or non-cardiac diseases causing MCs injury and cTns release [6,7,8,9,37,38,39,40,41,42,43]. For example, several pilot studies have clearly demonstrated the diagnostic value of hs-cTns in oral fluid in case of MI [8,37,38,39,40], in urine in case of diabetes mellitus (DM) [6], and in urine in case of arterial hypertension (AH) [7]. This may be of great clinical importance, since simple noninvasive and non-traumatic methods of obtaining urine and oral fluid do not require trained medical personnel during biomaterial acquisition, and it also reduces the risk of infection with hemocontact infections such as HIV and viral hepatitis, whereas, previously, the diagnostic value of cTns in this noninvasive-derived biomaterial was extremely low or questionable when moderate-sensitivity troponin immunoassays were used [8,9]. In my opinion, this is due to the fact that the concentration of cTn molecules in urine and/or oral fluid is relatively low and “not visible” to moderate sensitivity troponin immunoassays. In other words, the minimum detectable concentration of the moderate sensitivity immunoassays used was significantly higher than the concentration of cTn molecules present in urine and oral fluid. Thus, the diagnostic capabilities and diagnostic value of cTns in human body fluids have changed significantly due to the increased sensitivity of troponin immunoassays.

In addition to the increased diagnostic capabilities of hs-cTns, our understanding of the biology and metabolism of cTns has changed as troponin immunoassays have improved. For example, moderate-sensitivity troponin immunoassays, which have been widely used before the first high-sensitivity immunoassays, could not detect cTn molecules in blood serum and other body fluids in healthy people. Therefore, cTn molecules were considered as strictly intracellular molecules and the presence of cTns in blood serum was considered as one of the key pathological criteria to confirm MI in patients with clinical signs of myocardial ischemia [44,45,46,47,48]. In a number of cases, researchers noted that elevated levels could be observed in other severe pathological conditions (septic shock, massive PE, HF, Takotsubo CMP, acute myocarditis, and others), causing non-ischemic MCs injury. As a rule, such patients did not have complex clinical signs of myocardial ischemia (typical pain syndrome in the chest area, ischemic changes according to ECG and cardiac ultrasound), which helped doctors in differential diagnosis and in making a correct diagnosis [48,49,50]. However, due to the use of highly sensitive troponin immunoassays, cTn molecules began to be detected in the blood serum of all healthy patients [10,11,51,52]. These data allow one to consider cTn molecules as normal myocardial metabolites (provided that their level does not exceed the conventional 99th percentile values, which differ significantly in different troponin immunoassays). In addition, affected by the use of high-sensitivity troponin immunoassays, the researchers found that blood serum cTn concentrations were dependent on a number of biological features (gender, age and diurnal rhythm), the influence of which had never been traced before (when moderately sensitive immunoassays were used) [52,53,54,55,56,57]. However, the mechanisms of release of cTn molecules from MCs in healthy patients and the mechanisms of formation of these biological traits are not conclusively studied and require further clarification.

It is worth noting individually that the prevalence of elevated results and the extent of increase in cTn levels in many systemic and non-ischemic pathologies, as well as physiological conditions (physical exercise) is significantly higher with high sensitivity troponin immunoassays than with moderate sensitivity detection methods. This is confirmed by a number of comparative studies [58,59,60,61]. Thus, when using high-sensitivity immunoassays, differential diagnosis may be difficult and practitioners should be even more careful when interpreting elevated cTn levels in their patients. The upside of using hs-cTns in systemic and nonischemic pathologies is the ability to assess the short- and long-term prognosis of patients who suffer from these diseases.

Another promising area for the use of cTns in clinical practice is monitoring of the condition and assessment of CVD risk and complications in healthy patients or patients with certain risk factors (e.g., obesity, DM, AH, advanced age, etc.). High-sensitivity troponin immunoassays can detect insignificant levels of blood serum cTns, which may indicate subclinical myocardial injury [62,63,64,65,66]. For example, a study by Uçar et al. showed that in patients with newly diagnosed AH, elevated levels of hs-cTnT were associated with left ventricular (LV) hypertrophy and geometric parameters that were indicative of unfavorable LV remodeling [64]. McEvoy et al. also reported that baseline hs-cTnT levels were strongly associated with the development of LV hypertrophy and AH in the long term (over 6 years of follow-up). This can be used under outpatient treatment to identify those individuals who should be monitored more frequently and/or recommended necessary preventive measures [65]. In addition to that, Pattanshetty et al. reported that patients with AH-elevated levels of hs-cTnT are associated with a higher risk of major adverse cardiac and cerebrovascular events (MACCE) [66]. Thus, based on the results of a laboratory study, practitioners can identify patients who have a higher risk of developing CVD or the risk of developing complications in patients who have certain risk factors. This area for the potential use of hs-cTns has been actively studied recently. In the future, the data obtained will help to develop special algorithms for the diagnosis and monitoring of patients using high-sensitivity troponin immunoassays which can help physicians to initiate the earliest and the best therapeutic and preventive measures that will help improve patients’ prognosis. The possibility of using noninvasive-derived body fluids (e.g., urine), which are more convenient to obtain under outpatient treatment, adds special advantages to this process. For example, a recent study by Chen et al. shows that levels of hs-cTnI in the urine of diabetic patients can be a useful diagnostic tool for predicting cardiovascular complications. Thus, the researchers found that the concentration of hs-cTnI of more than 4.1 ng/L in urine is an independent factor for predicting adverse cardiovascular events in individuals with DM [6]. In acute conditions, urine is more difficult to obtain than blood. This is an important disadvantage of non-invasive biological fluids.

One of the important problems of laboratory methods for the determination of cTns in clinical practice is the lack of standardization [67,68,69,70]. This is expressed by the fact that if different immunoassays are used to determine cTns in the bodily fluids of patients, different concentrations of cTns will be obtained. Thus, there are now a large number of different methods for determining cTns in blood serum, and each of these methods produces different absolute values to make it impossible to compare the final results in the same sample. Moreover, serum cTn concentrations may differ by a factor of 2–10 or more during the use of different immunoassays [71,72,73]. This may be due to such factors as analytical characteristics (different sensitivity) and different diagnostic antibodies directed against different antigenic domains and epitopes (areas) of cTns molecule (anti-cTn). It should also be understood that cTn molecules in blood serum and probably in other body fluids (urine and oral fluid) are presented as a quite heterogeneous fraction: free whole molecules of cTnI and cTnT, binary complexes (cTnT + cTnI), triple complexes (cTnT + TnC + cTnI), and different fragments of cTnT and cTnI molecules with different sizes and molecular masses and modified forms (due to oxidation and phosphorylation) of the above cTn molecules [74,75,76,77,78,79]. Moreover, cTnT genes have several exons that can undergo alternative splicing [80,81]. So, it is theoretically possible to have more than 100 isoforms of cTnT that will have different amino acid sequences; consequently, their composition may have epitopes that will differ from the standard epitopes targeted by the diagnostic antibodies of the commercial kit [77,82,83,84,85]. This may result in some diagnostic antibodies not interacting with these epitopes in the immunoassay. In some particular cases, such as hereditary CMPs, mutations in the genes encoding cTns may occur [86,87,88]. Thus, changes at the nucleotide sequence level (DNA and mRNA of cTns) will subsequently be reflected as changes at the amino acid sequence level [89]. From a pathophysiological point of view, such amino acid changes of cTns can lead to the disruption of their function. This is the pathophysiological basis of CMPs. From a laboratory point of view, it can also lead to the disruption of antigen–antibody interactions (interaction of cTn molecules and anti-cTn antibodies) during immunoassay. Since the strength of the antigen–antibody interaction is directly proportional to the concentration of cTns, we can say that there are certain structural changes in cTns (in the region of those epitopes targeted by diagnostic antibodies) will disrupt the antigen–antibody interaction and thereby affect serum levels of cTns.

Different cTn molecules and their fragments, as well as oxidized and phosphorylated derivatives, may have different half-lives and different immune reactivities to diagnostic antibodies. This will have a significant impact on the diagnostic value of cTns and make an important contribution to the formation of differences in the laboratory examination of the body fluid of the patient [82,83,84,85]. In general, it can be noted that different troponin immunoassays in fact detect different cTn molecules in the same patient.

An additional factor that may influence the components of the heterogeneous cTns fraction and consequently the final assay result is the activity of enzymes causing proteolytic cleavage and modification (oxidation, phosphorylation, dephosphorylation) of cTn molecules and their fragments. For example, a number of studies have reported that phosphorylation of the cTnT molecule increases with LV hypertrophy and HF. From a pathophysiological point of view, this causes a decrease in myocardial contractility, and from the laboratory point of view, it may contribute to an underestimation of the cTn concentration, since the interaction of phosphorylated cTns with diagnostic antibodies of commercial kits is impaired. Thus, the detection of phosphorylated molecules and fragments of cTns requires special phosphorus-specific anti-cTn antibodies [90,91,92]. It is also quite interesting that the phosphorylated cTnT molecule is more sensitive to the proteolytic activity of the calpain enzyme compared to the unphosphorylated cTnT molecule [93]. The activity of proteolytic enzymes targets the peptide bonds connecting the aminoacid residues in the structure of cTn molecules. The disruption of the peptide bonds can lead to the formation of smaller fragments of cTns, which can change their physicochemical properties and affect the diagnostic value and diagnostic capabilities of cTns.

The elimination of cTns molecules plays an important role in the laboratory diagnosis of CVD, including MI, and this metabolic feature should be considered in the differential diagnosis.

Many metabolic features of cTns briefly discussed above are extremely understudied nowadays and their role in laboratory diagnosis and differential diagnosis of CVD requires clarification, generalization, and separate discussion of current knowledge on cTn metabolism which is the subject of the present article.

In general, the metabolic pathway of cTns can be divided into a number of stages (Table 1). Each of the stages may depend on a number of factors and have a major clinical significance.

Given the abundance of possible metabolic mechanisms and factors influencing cTn levels, as well as their potential clinical significance (Table 1), we should note the high relevance of further studies of cTns protein metabolism. In my opinion, this will optimize algorithms for early MI diagnosis, improve the differential diagnosis of MI and a number of CVDs, reduce the risks of misdiagnoses, expand the diagnostic capabilities of cTns in clinical practice and particularly develop algorithms for assessing the risk of CVD in healthy individuals, assess the prognosis of patients suffering from diseases causing myocardial injury, and validate methods for noninvasive diagnosis of CVD including MI.

## 2. Stage of Release of cTns Molecules from MCs into the Bloodstream

After the first high-sensitivity troponin immunoassays were developed in 2007–2010 and cTn concentrations were detected in a significant number of healthy individuals, many researchers asked: how are cTn molecules released from intact MCs into the blood serum? Researchers consider the following as hypothetical mechanisms: (1) apoptosis of MCs; (2) MCs regeneration and renewal; (3) increased permeability of cell membranes; (4) release of cTns by vesicular transport; (5) enhanced proteolytic degradation of cTns molecules inside the cell [94,95,96,97,98,99,100,101,102]. In addition, some of these mechanisms, such as apoptosis, can cause reversible and irreversible (under prolonged and/or excessive action of a damaging factor) MCs injury.

### 2.1. Significance of MCs Apoptosis in Release of cTns

As a result of the activation of the apoptosis processes in MCs, the activity of caspase enzymes increases. It can cleave the DNA and protein structures of the cell, but unlike necrosis, apoptosis preserves the integrity of the cell membrane for a relatively long time [95,96,102,103,104,105]. Modern methods for detecting apoptosis are light, electron, and fluorescent microscopy; flow cytometry; immunohistochemistry; as well as the TUNEL method, which is generally considered to be the most sensitive (early) and valuable criterion for detecting cell apoptosis. The TUNEL method allows researchers to visualize cell nuclei containing fragmented (influenced by caspases) DNA [106,107,108]. This method is actively used by researchers to study the causes and mechanisms of MCs apoptosis. A recent study by Weil et al. investigated the effect of short-term ischemia on MCs apoptosis in laboratory animals. The researchers simulated short-term ischemia (duration of ischemia was approximately 10 min) using balloon occlusion of the left anterior descending artery of the pig heart. The researchers used CAG to prove complete occlusion and restored normal coronary blood flow by deflating the balloon after 10-min ischemia. Then, myocardial histological examination was performed in one-half of these animals, and cTnI levels were examined in the other half over a 24-h period (First sampling was performed 10 min after reperfusion, and last sampling was performed 24 h after reperfusion). The histological examination of animal myocardium showed no signs of ischemic necrosis due to the short duration of ischemia. However, compared to the non-ischemic myocardial areas, the number of MCs in apoptosis (TUNEL-positive MCs) increased significantly (sixfold) in the focus of myocardium that underwent short-term ischemia. It is quite remarkable that cTnI levels began to rise as early as 10 min after reperfusion, and 30 min later, the cTnI levels reached the upper limit of a normal range (38 ng/L). After that, they continued to increase smoothly and reached extremely high values (1021 ± 574 ng/L) in 24 h after reperfusion [108]. This experimental work is of great value because it clearly demonstrates several key points: (1) short-term MCs ischemia triggers apoptosis processes, but does not cause cell necrosis; (2) apoptosis plays a significant role in the release of cTn molecules from myocardium into blood serum; (3) cTn levels in apoptosis begin to increase within the first minutes after reperfusion (in contrast to the atherothrombotic type of MI, when cTn molecules remain “blocked” in the ischemic zone and their time of penetration into the systemic bloodstream may significantly depend on the nature of reperfusion therapy and the phenomenon of “washout”); and (4) cTn levels in apoptosis can reach very high values, as in MI. However, it is worth noting the limitation of this study: the relatively short interval of myocardium study after reperfusion (24 h), and the results of this study cannot establish the reversibility of MCs injury in ischemia-induced apoptosis.

The literature also describes a lot of situations when MCs apoptosis occurs by other mechanisms (not related to short-term myocardial ischemia): with an increased LV myocardial preload and with myocardial tissue distension and increased neurohumoral stimulation through beta-adrenoreceptors [109,110,111,112,113,114,115,116,117].

According to the results of the experimental study, when LV myocardial preload increases, there is an increase in apoptosis and blood serum cTnI levels. Laboratory animals were injected intravenously with phenylephrine (injection rate = 300 μg of medication per minute) for one hour to increase end-diastolic pressure. After the experimental simulation, histological examination of the medications was performed in one half of the animals and blood for cTnI determination was taken from the other half of the animals. On histological examination of myocardium, the number of MCs in a state of apoptosis was significantly higher (*p* < 0.01) in the animals of the experimental group (31.3 ± 11.9 MCs/cm^2^) compared with the control group (4.6 ± 3.7 MCs/cm^2^). Twenty-four hours after the experimental simulation, the number of MCs undergoing apoptosis decreased significantly (6.2 ± 5.6 myocytes/cm^2^; *p* = 0.46), and no signs of MCs necrosis were observed at the indicated time intervals. At the same time, 30 min after the increase in end-diastolic pressure, serum cTnI levels exceeded the upper limit of normal range and after 1 h cTnI levels reached very high values (856 ± 956 ng/L) [118]. Overall, the results of this study regarding the important role of apoptosis in the release of cTn molecules add to the data described above.

Cheng et al. described the mechanisms of MCs apoptosis during myocardial distension [109]. Ventricular myocardial distension is noted in physiological conditions (long and strenuous exercise) and in a number of pathologies (AH, PE, chronic obstructive pulmonary disease, HF, etc.). Therefore, one can assume an important role of apoptosis in the release of cTn molecules from myocardium in these conditions. The extent of cTns release appears to depend on the strength and duration and the damaging factor. For example, in physical exercise, AH, and nonmassive PE, there is less stress on the ventricular myocardium. That is expressed by a relatively small increase in serum levels of cTns, whereas there is a sharp right ventricular myocardial overload in massive PE, which leads to a much more significant increase in cTns concentrations [119,120,121].

The effect of neurohumoral (adrenergic system) on the apoptosis processes was found in a study conducted by Singh et al. The opposite effects on apoptotic processes depending on the stimulation of β-adrenoreceptors were also noted: The stimulation of β1-adrenoreceptors increases apoptosis while the stimulation of β2-adrenoreceptors inhibits apoptosis [111,112,117]. The density of β-adrenoreceptor types changes in elderly patients. Particularly, the density of β2-adrenoreceptors decreases. This may lead to a decrease in the inhibitory effect on apoptosis and a relative increase in the activity of apoptosis mediated through β1-adrenoreceptors [113,114,115]. Thus, apoptosis may play a role in the release of cTn molecules from the myocardium in elderly patients. Several clinical studies using high-sensitivity troponin immunoassays have revealed age-related features of serum cTn levels, according to which cTn concentrations are higher in elderly people than in young people.

So, given the above data, there is every reason to suggest an important role of apoptosis in MCs injury and increased serum levels of cTns in PE, AH, HF, as well as prolonged and/or excessive exercise in the elderly, too. Further studies are needed to confirm and clarify the specific role of apoptosis in the release of cTn molecules from MCs.

### 2.2. MCs Regeneration and Renewal

Using labeled radioisotopes (14C) embedded in the DNA of MCs, some researchers have been able to reveal MC renewal (regeneration) and the fact that intensity of renewal decreases with age. Thus, 1% of MCs are renewed in a person aged 25 or less per year, while in a person aged 75 it is 0.45%. According to the authors, about half of human MCs are renewed during the whole life [122]. It is assumed that the process of MCs renewal is associated with the release of cTn molecules from the cytoplasm of MCs into the bloodstream, but it is still unknown how it occurs [122,123,124,125,126,127,128]. It is conceivable that cTns molecules would be released from progressively naturally aging and dying MCs. This mechanism may explain the presence of normal (less than the 99th percentile) serum levels of cTns determined by high sensitivity immunoassays in all healthy individuals. However, it is worth noting that the data on the presence of cardiac muscle tissue regeneration are contradictory and denied by a number of authors [129,130,131]. Therefore, additional studies are needed to validate this mechanism of cTns release.

### 2.3. Increased Permeability of the Cell Membrane of MCs

An increase in the permeability of a membrane of any human cell is associated with the release of cytoplasmic contents and particularly various molecules that can be used as specific biomarkers of certain diseases. The increased permeability of the cell membrane of MCs may develop as a result of two main mechanisms: (1) injury of cell membranes by proteolytic enzymes, the activity of which may increase already in short-term myocardial ischemia, and (2) as a result of myocardial tissue distension.

Short-term myocardial ischemia can occur in such conditions as strenuous exercise, psycho-emotional stress, sepsis (as a result of increased myocardial oxygen demand), and ischemic heart disease (due to reduced oxygen delivery to MCs). At the same time, the extent of cTns increase by this mechanism will depend on the strength of the physiological/pathological process. Thus, in minor or reversible (short-term) ischemia that develops during physical exercise and psychoemotional stress, the extent of cTns increase is less significant than in sepsis or MI. This is probably associated with the release of only cytoplasmic (free) fraction of cTns from MCs. The amount of this fraction of cTns is relatively small (about 3.5% of the total intracellular content of cTns), so the extent of the increase in these conditions will also be small. However, if the ischemia is more prolonged and severe (such as in severe sepsis or MI), the activity of proteolytic enzymes will be greater, leading to the cleavage of the structural fraction of cTns (proteins that are part of the troponin-tropomyosin complex) and consequently to a greater release of cTn molecules from MCs.

The second mechanism of cTns release is associated with an increase in myocardial load and distension. Researchers have established the relationship between myocardial overload and increased levels of cTns [9,132,133]. There is some similarity with the release of natriuretic peptides from the myocardium during myocardial distension which occurs during distension and overload of the heart muscle such as in HF. The extent of release of cTns and natriuretic peptide molecules from MCs in HF depends on the extent of distension and the stage of the pathological process. Therefore, patient prognosis is assessed by the level of these biomarkers [9]. The specific molecular mechanisms by which cTns are released from the myocardium during overload and distension are unknown. Some researchers believe that integrin molecules play one of the major roles in this mechanism. These are transmembrane glycoproteins that bind intracellular and extracellular space. The activation of these proteins is associated with myocardial distension. In their study, Hessel et al. demonstrated that activation of integrins leads to increased levels of cTnI. At the same time, cTn release was not associated with ischemic and necrotic changes in the myocardium because lactate and lactate dehydrogenase levels were normal and the histological pattern of myocardium in microscopy did not differ from controls [134].

### 2.4. cTns Release from MCs by Vesicular Transport

This possible mechanism of release of cTn molecules from cells has been described in experimental studies performed in vitro on isolated hepatocytes and MCs of laboratory animals. The researchers found that membrane vesicles are formed on the surface of hepatocytes and MCs. When MCs ischemia is induced, the number of membrane vesicles increases considerably [101]. It is assumed that these vesicles contain cytoplasmic proteins including molecules of cytoplasmic fraction of cTns. Thus, a small amount of the cytoplasmic fraction may be released into blood serum via a vesicular mechanism and the extent of molecule release increases with increasing ischemia, which agrees well with the concept of a biphasic increase in cTn levels observed during the development of MI. So, the first peak in the cTn concentration in MI is associated with the release of the entire cytoplasmic fraction of cTns, while the second peak of concentration is associated with the subsequent slower processes of MCs membrane disruption and proteolytic cleavage of the structural fraction of cTns which are part of the sarcomere. In the same physiological and some pathological conditions in which the damaging factor is removed (e.g., cessation of physical activity) only the cytoplasmic fraction of cTns is released as a result of vesicular transport. Therefore, the extent of the increase in lower serum levels is much less significant, and the duration of the circulation of increased levels is shorter accordingly.

### 2.5. cTn Molecules Proteolytic Degradation Processes

The size of a molecule is one of the key factors determining its capability to pass through the cell membrane. Thus, many low molecular mass compounds are much more intensively transported across the cell membrane. This is confirmed by the fact that the concentration of smaller protein molecules (e.g., myoglobin) increases considerably earlier in blood serum during the development of MI, while the levels of larger protein molecules (e.g., lactate dehydrogenase) increase considerably later [135,136]. The activity of intracellular proteolytic enzymes is the additional factor that can affect the rate and extent of release of a particular cardiac marker protein molecule. These enzymes cause the cleavage of cardiac marker molecules into smaller fragments, which can probably enable the latter to pass even through the intact cell membrane. The activity of certain proteolytic enzymes can be influenced by a number of factors such as increased myocardial load, changes in the acidity (pH) of intracellular environment intake of medications that can block or activate these enzymes and a number of other factors. For example, in the experiment by Feng et al., it was found that increased LV myocardial preload increases the activation of the intracellular enzyme calpain which enhances the proteolytic cleavage of cTnI. This can lead to an increase in its concentration in blood serum. In this experiment, the increased activation of calpain and increased cleavage of cTnI were not associated with myocardial ischemia. This was confirmed by normal lactate levels. After the elimination of experimentally induced myocardial overload, the researchers noted a decrease in calpain activity, a decrease in cTnI degradation and normalization of LV functional activity. Similar changes were also noted with calpeptin administration, which is a specific inhibitor of calpain [102]. In addition to the role of this mechanism in the release of cTnI molecules from the myocardium an important pathophysiological significance can be noted. Since cTnI plays an important role in the regulation of myocardial contraction and relaxation, the cleavage of this protein by calpain can further impair myocardial function. This would contribute to the pathogenesis of HF. Thus, calpain can be considered as a target for the development of therapeutic agents that could potentially have important clinical significance.

Another interesting mechanism by which the proteolytic cleavage activity of cTns is enhanced involves changes in the acidity (pH) of the intracellular environment [137,138]. MCs together with liver and kidney cells maintain the acid–base balance of the human body. Their important function is the utilization of lactate which is a product of anaerobic metabolism of other cells, such as red blood cells, myosymplasts of skeletal muscle tissue, etc. During normal metabolism, lactate enters hepatocytes, as well as myocardial and renal cells, where it is converted to pyruvate by the enzyme lactate dehydrogenase, which can then be converted to glucose (Cori cycle) by gluconeogenesis. Some pyruvate can be converted into acetyl coenzyme A, which will be further metabolized in mitochondria in the tricarboxylic acid cycle (Krebs cycle) [137,139]. However, this pathway (aerobic pathway) depends on oxygen and its functioning may be impaired under conditions of myocardial ischemia. As a result of myocardial ischemia, Krebs cycle functioning is impaired and MCs will be forced to switch to anaerobic metabolism. This process will be accompanied by increased lactate formation [139,140,141]. Thus, under conditions of MCs ischemia, the acid–base equilibrium will be considerably disturbed. The accumulation of lactate in MCs will lead to the acidosis of the intracellular environment, which is a trigger mechanism for the activation of several proteolytic enzymes (e.g., matrix metalloproteinases) and apoptotic enzymes (e.g., caspases) which can cleave cTn molecules and contribute to the release of reduced cTn fragments from MCs into blood serum. It should also be noted that with the marked activation of proteolytic and apoptotic enzymes, in addition to the cleavage of cTn molecules, there is likely to be cleavage of protein molecules that are part of the cell membrane. Due to this fact, membrane permeability will increase and further contribute to the release of cTns. Thus, the mechanism of the proteolytic cleavage of cTns is closely related to the mechanism of increased membrane permeability. The severity of pathological processes will be related to the activity of these two mechanisms and consequently to the extent of increase in cTn levels. For example, during physical exercise and psycho-emotional stress the extent of increase in cTns levels is relatively small, which suggests subclinical and reversible injury to MCs without further consequences significant for the morphology and function of the heart muscle. The data of magnetic resonance imaging with contrasting gadolinium preparations support this finding. Thus, no signs of necrosis and cardiosclerosis were detected in athletes [142]. Nevertheless, regular myocardial injury as even a result of physiological factors (e.g., physical loads, stressful situations) can be dangerous for human health as noted in the works of several researchers [143,144,145,146,147,148]. When using hs-cTns, the possibility of cTns elevation in these physiological conditions should be taken into account and should be clarified in patients while taking their history in order to reduce the risk of MI overdiagnosis [149,150].

## 3. Extracardiac Expression of cTn Molecules and cTn Release from Skeletal Muscle Tissues

Extracardiac expression is worth noting as another possible but most controversial mechanism of increased cTn levels. According to this mechanism, there is a possibility of the expression of cTn molecules in skeletal muscle tissue in the case of CKD and a number of hereditary myopathies. Thus, several independent research groups have used polymerase chain reaction and Western blotting to detect cTnT protein molecules and cTnT informational RNA in the skeletal muscle of patients suffering from end-stage CKD [151]. However, it has not yet been scientifically proven that the cTnT molecules expressed in skeletal muscle can be released into the bloodstream in amounts sufficient for their detection by troponin immunoassays. Two research groups led by Messner [152] and Jaffe [153] found evidence of cTnT expression in the skeletal muscles of patients who suffered from various myopathies (Duchenne muscular dystrophy, sarcoglycanopathy, facioscapulohumeral muscular dystrophy, and other hereditary myopathies). The researchers found no evidence of CVD but noted elevated serum levels of cTns in these patients [152,153]. This suggests that cTns expressed in skeletal muscle can be released into blood serum and cause elevated cTn concentrations in blood serum. Thus, to avoid diagnostic errors, practitioners and researchers should take into account the possibility of extracardiac expression of cTnT and nonspecific increases in concentration in some inherited skeletal muscle pathologies. However, other investigators in their manuscript have not confirmed the presence of cTn expression in skeletal muscles in case of hereditary myopathies and believe that elevated cTn levels in skeletal muscle diseases are only due to false-positive reactions [154,155]—the interactions of anti-cTn antibodies with skeletal Tn molecules released from myosymplasts during their alteration.

In general, the possible mechanisms of cTn release are presented in Table 2.

## 4. The Role of Diurnal Rhythm in the Release of cTn Molecules from MCs

In a number of recent studies researchers have detected diurnal rhythm in cTns levels using high-sensitivity troponin immunoassays. Thus, it has been shown that cTnT levels are the highest in the morning hours and then gradually decrease and reach their minimum in the evening and night hours [156,157,158,159,160]. One study by van der Linden et al. reported that diurnal rhythm of cTnT may influence early diagnostic algorithms for MI. The authors noted that the maximum fluctuation of serum TnT levels over an entire day was about 50 ng/L and about 20 ng/L during 1 hour [158]. In modern diagnostic algorithms for NSTEMI, small increases in cTnT concentrations can play an important role. Thus, an increase in the cTnT concentration during the first and second hour by over 5 and 10 ng/L, respectively, indicates a high probability of NSTEMI [12,161]. Therefore, the natural diurnal rhythm of cTnT can be interpreted as pathological and lead to overdiagnosis of MI. cTnI is not characterized by diurnal rhythm. It has also been reported in several studies.

The mechanisms for the formation of diurnal rhythm of cTnT may be closely related to changes in the functional activity of those systems of the human body that have a relatively negative effect on the cardiovascular system. For example, in the morning there is an increased activity of the hemostasis system, as well as of the sympathoadrenal, renin-angiotensin-aldosterone and pituitary-thyroid systems. This is expressed by the increased concentration of compounds such as prothrombin, catecholamines, cortisol, and thyroid hormones and other components in blood [162,163,164,165,166]. These molecules can have a great impact upon hemodynamics, blood pressure, and heart rate and also contribute to the release of cTns. For example, an increase in blood pressure can increase the load on the ventricular myocardium and thereby contribute to the activation of proteolytic cleavage of cTns in MCs and increase the permeability of the plasma membrane of MCs. Increased heart rate causes increased oxygen demand for myocardium, as well as relative ischemia of MCs. This may initiate apoptotic and proteolytic enzymes that will contribute to the release of cTns by a number of mechanisms that are described in this manuscript above.

In addition, some biochemical differences between cTnT and cTnI may play an important role. For example, the cytoplasmic fraction of cTnT is higher than that of cTnI (approximately 6–7% vs. 3–4%). Thus, the possibility of releasing the cytoplasmic fraction under any natural (physiological) conditions is higher for cTnT than for cTnI [167]. Other biochemical and laboratory features of cTns are summarized in the Table 3.

It is worth noting that there is very little research devoted to the study of diurnal rhythms, and an important limitation of this research is the small selection of patients. In addition, diurnal rhythms have not been proven for all existing high-sensitivity troponin immunoassays, and no such studies have been performed for some immunoassays. Additional clarifying studies on larger selections for all of the high sensitivity troponin immunoassays currently used in clinical practice are required for validation.

## 5. Conclusions and Future Perspectives

On the basis of this narrative review, I can conclude about the important role of cTns metabolism in the diagnosis of CVD including MI: differential diagnosis of CVD and several extracardiac diseases that cause an increase in cTn levels or affect the circulation and elimination of cTns from the bloodstream. However, it should be noted that existing data on cTns metabolism are extremely scarce, and further research is required. There is a need to focus on studying the specific mechanisms of cTns release and to establish their exact role in cTn release in certain diseases and physiological conditions. So, today, the only established mechanism for increasing cTns in humans is myocardial necrosis. All other mechanisms are hypothetical and very difficult to prove in humans. The role of biological factors (gender, diurnal rhythm, and age) in the release of cTns molecules should be finally clarified. It should also be noted that biological and analytical variations of cTns are clinically less relevant if concentrations are significantly elevated. However, this is a serious issue if cTn levels are moderately elevated, for example, in the early stages of MI and may complicate diagnosis.

## Figures and Tables

**Table 1:** Metabolic pathway of cTns.

Main Stages of Metabolic Pathway of cTns	Brief Description of the Stage and Factors That Affect Metabolic Pathway of cTns	Main Clinical and Diagnostic Significance of Metabolic Pathway of cTns
Stage of biosynthesis and release of cTns molecules from MCs into the bloodstream	cTn molecules are mainly synthesized in MCs and can be released into blood serum both under physiological conditions (physical and psychoemotional stress) and during pathological processes (e.g., myocarditis, sepsis, hypertensive crisis, PE, Takotsubo syndrome, and a number of others). Using high-sensitivity immunoassays, it has also been found that the degree of release of cTns molecules from cells into the bloodstream depends on several biological factors: (1) gender, (2) diurnal rhythm, and (3) age-related.	The extent of release of cTn molecules from the myocardium may depend on the type and severity of the pathological process or physical load (under physiological conditions). Gender peculiarities have an important clinical significance in modern diagnostic algorithms which are used in some algorithms of early diagnosis of MI. Diurnal rhythm and age-specific features are not yet reflected in the current clinical guidelines and diagnostic algorithms due to little study.
The stage of cTns molecules’ circulation in blood serum	The molecules circulating in blood serum can be affected by a number of enzymes belonging to the groups of proteases, kinases, phosphatases, and oxidases. The activity of these enzymes may change under physiological and pathological conditions, as well as medication.	Concentration of cTns in blood serum may depend on the activity of a number of enzymes (proteases, kinases (phosphorylases), phosphatases, and oxidases) that cleave and modify cTn molecules, leading to changes in the antigen–antibody interaction in immunochemical assays.
The stage of cTns molecules’ elimination from blood serum	Elimination of cTns molecules from blood serum can be accomplished by the following mechanisms: (1) elimination of molecules through hematotissue barriers; (2) uptake of cTn molecules by cells of reticuloendothelial system and intracellular cleavage into amino acids within these cells; (3) cleavage of cTn molecules in bloodstream as a result of proteolytic enzymes.	cTns concentration in blood serum has inverse dependence on elimination rate. Thus, when the rate/extent of elimination of cTns molecules from the bloodstream decreases, there will be an increase levels of cTns in blood serum.

**Table 2:** Possible mechanisms of cTns release from MCs.

Mechanism of cTns Release	Brief Description of the Mechanism	Literature Source
MCs necrosis	Cell necrosis is accompanied by destruction of the cell membrane. This will contribute to the release of all cytoplasmic components from the cell into the blood serum.	[4,9,11]
MCs apoptosis	MCs apoptosis develops as a result of several factors (short-term myocardial ischemia, myocardial distension, increased activity of neurohumoral (sympathoadrenal) system) and may be accompanied by a significant increase in cTn levels.	[95,96,102,103,104,105]
MCs regeneration and renewal	According to some researchers, a small part of MCs can be renewed (replaced). Gradual death of senescent MCs may result in the release of small amounts of cTn molecules into blood serum.	[96,122,123,124]
Increased permeability of the cell membrane of MCs	The extent of cell membrane permeability is an important factor that determines whether intracellular molecules can be released from the cell to the outside.	[132,133,134]
cTns release from MCs by vesicular transport	According to this mechanism, cTn molecules can escape outside the cells as part of the membrane vesicles.	[101]
cTns molecules proteolytic degradation processes	The size of the molecule is considered as a factor influencing its capability to be released through the cell membrane; smaller molecules are released earlier and faster compared to larger molecules. A number of proteolytic enzymes (calpain, matrix metalloproteinases) can be activated under certain physiological and pathological conditions and catalyze the degradation of cTn molecules into small fragments that will contribute to their passage through the plasma membrane.	[102]
Extracardiac expression of cTn molecules and troponin release from skeletal muscle tissues	According to several authors, cTn molecules can be expressed in skeletal muscle of patients under certain conditions (CKD, inherited myopathies) and then released from skeletal muscle, causing increased cTn levels in blood serum.	[151,152,153]

**Table 3:** Comparative biochemical characteristics of cTns.

Clinical and Laboratory Characteristics	cTnI	cTnT
Molecular weight, kilodaltons	23	36
Specificity	Absolute (only myocardial expression)	Not absolute (there is conflicting information about the expression of cTnT in skeletal muscles)
Myocardial content (mg per 1 g of myocardial mass)	4–6	10–11
Volume of cytosolic fraction	3–4%	6–7%
Standardization of highly sensitive detection methods	Poor (there are many manufacturers of analysis methods for determining hs-cTnI)	Good (there is only 1 manufacturer of methods for the analysis of hs-cTnT)

## Data Availability

Not applicable.
